# Surgeon’s experience level and risk of reoperation after hip fracture surgery: an observational study on 30,945 patients in the Norwegian Hip Fracture Register 2011–2015

**DOI:** 10.1080/17453674.2018.1481588

**Published:** 2018-06-04

**Authors:** Ane L Authen, Eva Dybvik, Ove Furnes, Jan-Erik Gjertsen

**Affiliations:** 1 Department of Clinical Medicine, University of Bergen, Bergen;;; 2 Norwegian Hip Fracture Register, Department of Orthopaedic Surgery, Haukeland University Hospital, Bergen, Norway

Background and purpose — In Norway, surgeons with variable levels of surgical experience manage hip fractures. We investigated whether the experience level of the surgeon affected the risk of reoperation after hip fracture surgery.

Patients and methods — This is an observational register-based study of 30,945 hip fractures reported to the Norwegian Hip Fracture Register in the period 2011–2015. An experienced surgeon was defined as a surgeon with more than 3 years’ experience in hip fracture treatment. If more than 1 surgeon performed the procedure, the most experienced surgeon defined the level of experience. Relative risks of reoperations were calculated with Cox regression analyses with adjustments for age groups, sex, and ASA class.

Results — Overall, patients operated by an inexperienced surgeon had a higher risk of reoperation compared with an experienced surgeon (RR =1.2 (95% CI 1.1–1.4)). Displaced femoral neck fractures (FNFs) had higher risk of reoperation regardless of operation method when managed by an inexperienced surgeon compared with an experienced surgeon (RR =1.7 (95% CI 1.4–2.1)). Sub-analyses of other fracture types and operation methods showed no statistically significant differences between the 2 groups of experience.

Interpretation — FNFs operated by surgeons with less than 3 years’ experience in fracture treatment had a small increased risk of reoperation. The study indicates that experienced surgeons should manage displaced FNFs, and fractures operated with hemiprosthesis.

## 

Approximately 9,000 hip fractures are operated annually in Norway. Roughly 25% of these patients die within the first year after the fracture (Figved et al. 2009, Gjertsen et al. 2010). It is therefore important to optimize the treatment and decrease the risk of reoperation in these patients.

Surgery for a hip fracture is most often done during the daytime, often by a consultant surgeon assisting a resident surgeon. In some cases, however, the resident surgeon operates without the assistance of a more experienced colleague. There are no guidelines in Norway as to whether hip fracture operations should be managed by surgeons that are more experienced and the practice differs from hospital to hospital.

Earlier studies indicate a direct relationship between surgeon volume and outcome in different surgical disciplines (Figved et al. 2009, Aquina et al. 2015, Damle et al. 2016, Kelly et al. 2016). Significant coherence has also been demonstrated between surgeon volume and outcome (Browne et al. 2009). 1 large register study reported that interns and junior residents performed half of all fracture-related surgery and that one-third of primary operations performed by junior residents were unsupervised (Andersen et al. 2014). Some studies have addressed surgeon experience in regards to the outcome after hip fracture surgery, though the results are ambiguous. 1 clinical study reported a higher reoperation rate among so-called demanding hip fracture procedures, if residents had not been supervised (Palm et al. 2007). Another study found no statistically significant difference between residents and consultant surgeons in reoperation rates, but higher mortality after procedures performed by residents (Khunda et al. 2013).

The Norwegian Hip Fracture Register (NHFR) has since 2005 collected nationwide information on all hip fractures in Norway (Gjertsen et al. 2008). In the present study, we used data from the NHFR to investigate whether there were any differences in reoperation rates between hip fracture operations performed by an experienced surgeon compared with an inexperienced surgeon.

## Patients and method

### The Norwegian Hip Fracture Register (NHFR)

Since 2005, surgeons managing hip fractures have voluntarily reported all hip fracture operations to the NHFR on a standardized 1-page questionnaire. Patient information registered includes age, sex, ASA classification, cognitive function, and national ID number (Gjertsen et al. 2008). A reoperation was defined as any secondary operation performed due to complications after the primary operation including both major reoperations such as re-osteosynthesis or secondary prosthesis and minor reoperations such as removal of implants, soft tissue debridement for infection, and closed reduction of a dislocated prosthesis. The national ID number allows link of an eventual reoperation to the former operation and linkage to death and emigration by Statistics Norway. In this way, the NHFR monitors the outcome of the operation. Furthermore, classification of the fracture, type of operation, cause and type of reoperation, information on implants, and duration of the procedure is registered.

The surgeons classified the intracapsular femoral neck fractures (FNFs) as undisplaced (Garden 1 or 2) or displaced (Garden 3 or 4). Extracapsular fractures were divided into basocervical FNFs, trochanteric fractures, and subtrochanteric fractures. The trochanteric fractures were further classified into 2-part trochanteric fractures (AO/OTA A1), multi-fragment trochanteric fractures (AO/OTA A2) or intertrochanteric fractures (AO/OTA A3). In 2011, information on surgeon’s experience was added to the questionnaire. Surgeon’s experience in fracture surgery is classified into more or less than 3 years. The question regarding surgeon’s experience is answered by the operating surgeon after each operation. The name and position of the surgeon is not registered in the database. There is no information on the number of procedures performed by the individual surgeon and each surgeon’s experience level is defined by number of years performing fracture surgery. If more than 1 surgeon performed the procedure, the most experienced surgeon defined the level of experience. For the time-period 2008–2014, a completeness analysis of the NHFR was conducted by comparing the registry with the Norwegian patient registry (NPR) (Havelin et al. 2015, 2016). Completeness of primary operations in the NHFR was 91–94% for hemiprosthesis and 80–86% for osteosynthesis. The completeness for reoperations after hemiarthroplasty and osteosynthesis has been found to be 68% and 65% respectively when compared with the NPR (Furnes et al. 2017).

### Data and study sample

**Figure 1.**
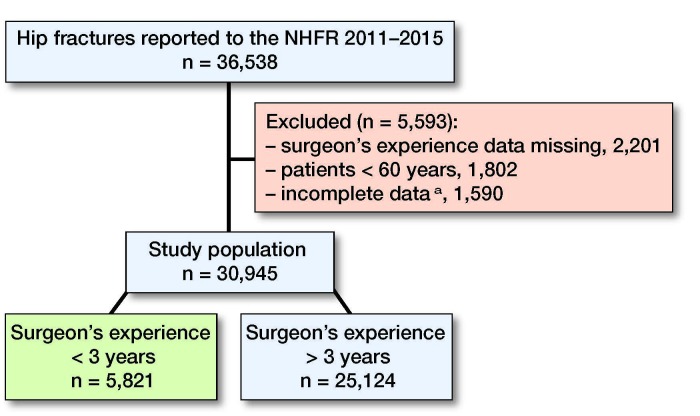
Description of selection of patient study group from the NHFR. **^a^**Incomplete data on fracture type, operation type, ASA classification or operation time.

Between January 1, 2011 and December 31, 2015, 36,538 hip fractures were reported to the NHFR on the new questionnaire including data on surgeons’ experience level. 30,945 cases were eligible for the study (Figure 1).

### Statistics

The Pearson chi-square test was used to compare categorical variables in the independent groups. Student’s t-test was used to compare continuous variables. The significance level was set at 0.05. Cox survival analyses were done to calculate risk of reoperations and risk of death. In all regression models surgical experience >3 years was used as the reference. 95% confidence intervals (CIs) were calculated for relative risks. Adjustments were done for age groups, sex, and ASA class. The proportional hazards assumption was investigated visually by use of log-minus-log plots. For displaced femoral neck fractures treated with hemiarthroplasty the curves crossed each other after 60 days. We therefore performed additional Cox regression analyses with the follow-up divided into 2 time-periods. The first period ran from the day of surgery until 60 days postoperatively and the second period commenced at 60 days and ran until December 31, 2015. The proportional hazard was fulfilled within the 2 time-periods. Sub-analyses were done for different fracture types and operation methods. Further, the Cox model was used to construct survival curves for all fractures and for displaced femoral neck fractures with adjustments for age group, physical status (ASA score), and sex. Adjustments for patients operated on both sides were not done, since an earlier study has shown that this will not alter the conclusion for the entered covariates (Lie et al. 2004). The analyses were performed using IBM-SPSS version 24 software (IBM Corp, Armonk, NY, USA) and the cmprsk Library in the statistical package “R” (https://cran.r-project.org/Package=cmprsk).

### Ethics, funding, and potential conflicts of interest

The NHFR has permission from the Norwegian Data Inspectorate to collect patient data based on written consent from the patients. (Permission issued January 3, 2005; reference Number 2004/1658-2 SVE/-). Informed consent from patients is entered in the medical records at each hospital. The Norwegian Hip Fracture Register is financed by the Western Norway Regional Health Authority (Helse-Vest). No competing interests declared.

## Results

### Baseline characteristics

The average age of the patients was the same, 83 years, for those operated by experienced surgeons and for those operated by inexperienced surgeons. Patients operated by experienced surgeons had statistically significantly more comorbidity than patients operated by inexperienced surgeons (ASA class 3–5: 66% vs. 62%, respectively) (Table 1).

**Table 1.** Characteristics of all patients according to surgeon’s experience

Factor	< 3 years	> 3 years	p-value
Total number	5,821	25,124	
Mean age (SD) at fracture	83 (8.6)	83 (8.5)	0.08 ^a^
Women, n (%)	4,113 (71)	17,625 (70)	0.4 ^b^
ASA score, n (%):			< 0.001 ^b^
ASA 1–2	2,239 (39)	8,605 (34)	
ASA 3–5	3,528 (62)	16,519 (66)	
Operation time according to fracture type, minutes (SD):			
All fractures	57 (28)	64 (32)	< 0.001 ^a^
Undisplaced FNFs	32 (18)	32 (23)	0.3 ^a^
Displaced FNFs	69 (29)	72 (26)	< 0.001 ^a^
Trochanteric 2-fragmented	58 (21)	49 (22)	< 0.001 ^a^
Trochanteric multi-fragmented	63 (25)	60 (30)	< 0.001 ^a^
Inter- /subtrochanteric	79 (32)	91 (41)	< 0.001 ^a^
Operation time according to type of operation, minutes (SD):			
Screw osteosynthesis	30 (13)	25 (13)	< 0.001 ^a^
Hemiarthroplasty	78 (25)	76 (25)	0.06 ^a^
Sliding hip screw	62 (23)	60 (30)	0.001 ^a^
Long intramedullary nail	86 (36)	93 (41)	0.01 ^a^

ASA: American Society of Anesthesiologists.

FNF: femoral neck fracture.

^a^Independent samples t-test.

^b^Pearson chi-square test.

The patients were divided into subgroups to investigate the risk of reoperation for the different fracture types and operation methods (Table 2).

**Table 2.** Characteristics of patients according to fracture type

Factor	Undisplaced FNFs	Displaced FNFs	Trochanteric 2-fragmented	Trochanteric multifragmented	Intertrochanteric/ subtrochanteric
Total number	4,220	13,098	5,077	5,000	2,303
Mean age at fracture	80	83	83	84	83
Women, n (%)	2,292 (69)	9,023 (69)	3,554 (70)	3,750 (75)	1,750 (76)
ASA score, n (%):					
ASA 1–2	1,730 (41)	4,453 (34)	1,777 (35)	1,700 (34)	806 (35)
ASA 3–5	2,490 (59)	8,645 (66)	3,300 (65)	3,300 (66)	1,497 (65)
Duration of surgery, minutes	32	72	52	61	89
Experienced surgeons, n (%)	2,954 (70)	11,919 (91)	3,503 (69)	3,750 (75)	2,027 (88)
Median follow-up, years	1.4	1.3	1.2	1.2	1.2

FNF: femoral neck fracture.

### Fraction of operations performed by inexperienced surgeons

**Figure 2.**
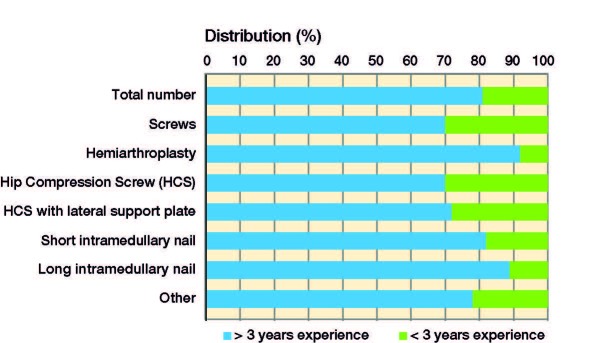
Proportion of procedures performed by experienced surgeons and inexperienced surgeons.

Experienced surgeons participated in 25,124 (81%) of the total number of 30,945 hip fracture operations. The proportion of experienced surgeons for the different surgical methods was 70–92% (Figure 2). The highest fraction of experienced surgeons was found for hemiarthroplasties and operations with a long intramedullary (IM) nail. Screw osteosyntheses and operations with a hip compression screw were the procedures most likely to be performed by inexperienced surgeons.

### Change in experience level in the period 2011–2015

**Figure 3.**
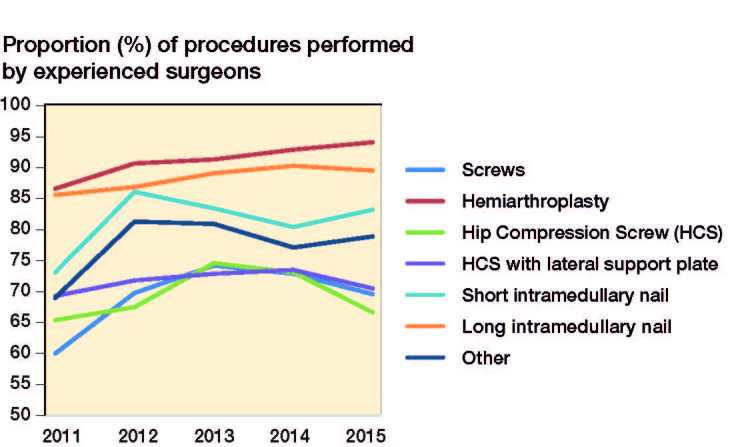
Changes in proportion of procedures performed by experienced surgeons.

In the period 2011–2015, an increasing proportion of operations was performed by experienced surgeons (Figure 3). This tendency was present for almost all operation methods. However, for osteosyntheses with hip compression screw with/without lateral support plate the proportion of procedures performed by experienced surgeons increased from 2011 to 2013, but decreased again from 2013 to 2015 (Figure 3).

### Duration of surgery

The duration of surgery was longer for displaced femoral neck fractures and sub-/intertrochanteric fractures and shorter for 2-fragmented and multi-fragmented trochanteric fractures when the operation was performed by an experienced surgeon (Table 1). Further, duration of surgery was longer for IM nails and shorter for screw osteosyntheses and sliding hip screws when the operation was performed by an experienced surgeon (Table 1).

### Reoperations

**Figure 4.**
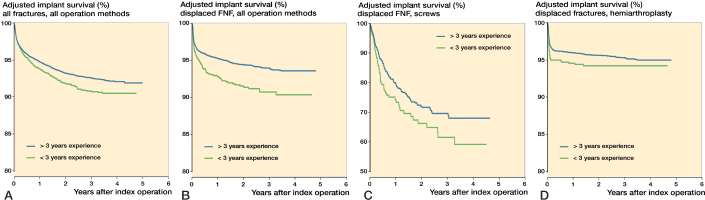
Cox regression curves for implant survival after different fracture types and operation methods adjusted for age groups, gender, and ASA class. A. All fractures/all operations methods (RR =1.2 (1.1–1.4), p = 0.001). B. Displaced femoral neck fractures/all operation methods (RR =1.7 (1.4–2.1), p < 0.001). C. Displaced femoral neck fractures/screw osteosynthesis (RR =1.4 (1.0–1.9), p = 0.001). D. Displaced fractures/hemiprosthesis (RR =1.3 (0.99–1.8), p = 0.06).

There was an increased risk of reoperation for patients operated by an inexperienced surgeon compared with patients operated by an experienced surgeon (5.3% vs. 4.2%, RR =1.2 (CI 1.1–1.4)) (Table 3 and Figure 4a).

**Table 3.** Reoperation risk for surgeon experience level: all fracture types/all operation methods

Operation method	< 3 years’ experience		> 3 years’ experience		RR ^a^	95% CI	p-value ^b^
	Total	Reop. (%)	Total	Reop. (%)			
All fracture types:							
All operation methods	5,821	403 (6.9)	25,124	1,411 (5.6)	1.2	1.1–1.4	0.001
Hemiarthroplasty	1045	53 (5.1)	11,619	470 (4.0)	1.3	0.95–1.7	0.1
Screw osteosynthesis	1,379	194 (14)	3,209	415 (13)	1.1	0.94–1.3	0.2
Hip compression screw	2,619	115 (4.4)	6,257	325 (5.2)	0.87	0.70–1.1	0.2
Short intramedullary nail	508	21 (4.1)	2,329	100 (4.3)	0.94	0.59–1.5	0.8
Long intramedullary nail	168	9 (5.4)	1,343	67 (5.0)	1.04	0.52–2.1	0.9
Undisplaced FNFs:							
All operation methods	1,267	141 (11)	2,953	296 (10)	1.1	0.92–1.4	0.2
Screw osteosynthesis	1,178	139 (12)	2,519	273(11)	1.1	0.91–1.4	0.3
Hemiprosthesis	47	0 (0)	334	16 (4.8)	^c^	^c^	^c^
Displaced FNFs:							
All operation methods	1,206	109 (9.0)	11,892	608 (5.1)	1.7	1.4–2.1	< 0.001
Screw osteosynthesis	198	54 (27)	675	137 (20)	1.4	1.0–1.9	0.04
Hemiprosthesis	982	52(5.3)	11,046	442 (4.0)	1.3	0.99–1.8	0.06
Trochanteric 2-fragmented fractures:							
All operation methods	1,581	41 (2.6)	3,496	93 (2.7)	0.97	0.67–1.4	0.9
Hip compression screw	1,278	30 (2.3)	2,482	62 (2.5)	0.95	0.61–1.5	0.8
Intramedullary nail	271	8 (3.0)	948	28 (3.0)	0.95	0.43–2.1	0.9
Trochanteric multifragmented fractures:							
All operation methods	1,229	65 (5.3)	3,771	198 (5.3)	1.0	0.77–1.4	0.9
Hip compression screw	949	53 (5.6)	2,394	127 (5.3)	1.1	0.80–1.5	0.6
Intramedullary nail	264	11 (4.2)	1,290	65 (5.0)	0.81	0.43–1.5	0.5
Intertrochanteric and subtrochanteric fractures:							
All operation methods	287	24 (8.4)	2,016	127 (6.3)	1.3	0.87–2.1	0.2
Hip compression screw	173	17 (9.8)	767	68 (8.9)	1.1	0.62–1.8	0.8
Intramedullary nail	109	7 (6.4)	1,219	57 (4.8)	1.5	0.67–3.3	0.3

FNF: femoral neck fracture.

^a^RR relative risk for reoperation. Surgeon’s experience >3 years set to 1.

^b^Cox regression analyses with adjustments for age group, gender, and ASA class.

^c^RR could not be calculated due to no reoperations.

For undisplaced femoral neck fractures (FNFs) the risk of reoperation was similar between inexperienced and experienced surgeons (Table 3). Also, when performing sub-analyses including only screw osteosynthesis for those fractures, reoperation rates due to failure of the osteosynthesis (RR =0.93, p = 0.7) or due to avascular necrosis of the femoral head (RR =1.3, p = 0.3) were similar between inexperienced and experienced surgeons There were no reoperations after hemiprosthesis for undisplaced femoral neck fractures performed by inexperienced surgeons. Accordingly, it was not possible to calculate risk estimates for this group.

Some fracture types and certain operation methods had a higher risk of reoperation after operation by inexperienced surgeons (Table 3). For displaced FNFs there was a higher risk of reoperation after operations performed by inexperienced surgeons (RR =1.7 (CI 1.4–2.1)) (Table 3 and Figure 4b). Sub-analysis showed that displaced FNFs operated with screw osteosynthesis by inexperienced surgeons had a higher risk of reoperation (RR =1.4 (CI 1.0–1.9)) (Table 3 and Figure 4c). Further, displaced FNFs operated with hemiprosthesis had more reoperations after operation by an inexperienced surgeon when analyzing the whole study period, but the difference was not statistically significant (RR =1.3 (CI 0.99–1.8)) (Table 3 and Figure 4d). However, there was an increased risk of reoperation in the first 60 days after hemiarthroplasty for displaced femoral neck fracture performed by inexperienced surgeons (RR =1.5 (CI 1.1–2.0)). After 60 days no statistically significant difference could be found (RR =0.64 (CI 0.20–2.1)). There was an increased risk of reoperation due to dislocation in the first 60 days after hemiprosthesis operation by an inexperienced surgeon (RR =2.0 (CI 1.1–3.9)). There was no difference in risk of reoperation due to infection after hemiprosthesis performed by inexperienced surgeons compared with experienced surgeons (RR =1.1, p = 0.8).

For other fracture types and operation methods no statistically significant differences in risk of reoperation could be found between inexperienced and experienced surgeons (see Table 3).

When performing analyses with adjustments also for duration of surgery similar results were found.

### Mortality

30-day mortality was 8.2% after operation by an experienced surgeon and 7.9% after operation by an inexperienced surgeon (RR =0.98 (CI 0.89–1.1)). 1-year mortality was 24% after surgery performed by an experienced surgeon and 25% after operation by an inexperienced surgeon (RR =1.0 (CI 0.99–1.1)).

## Discussion

We found statistically significant more reoperations after hip fracture operations performed by inexperienced surgeons compared with experienced surgeons, which was also the case in some subgroups of fractures and surgical methods. Inexperienced surgeons could perform operations for undisplaced FNFs without an increased risk of reoperation. On the other hand, displaced FNFs treated with screw osteosynthesis by inexperienced surgeons resulted in an increased risk of reoperation. Further, displaced FNFs operated with hemiarthroplasty by inexperienced surgeons showed an increased risk for both reoperation of any cause and reoperation due to dislocation the first 60 days postoperatively.

Findings in an observational, register-based study are less conclusive then those from randomized clinical trials. However, if confounders are corrected for, the level of evidence in validated national registry studies should be respected, reflecting the actual real-life national status. A considerable strength of this study was the large study sample, including more than 30,000 hip fractures from a whole country. Thus, the external validity is high.

Another strength of this study is the high reporting rate to the NHFR by Norwegian hospitals. When compared with the Norwegian Patient Register (NPR), approximately 90% of all hip fractures operations performed in Norway during the period 2011–2014 were registered in the NHFR (Havelin et al. 2016). Registration of reoperations, on the other hand, had a lower completeness, which gives an inaccurate estimation of the risk for reoperation in total. When compared with the NPR, 70% of reoperations were registered in the NHFR (Furnes et al. 2017). This annual report suggests that there is some degree of uncertainty connected to these registrations. However, we have no indication of systematic underreporting, or that the completeness of reporting of reoperations is different in the 2 groups compared, so the relative risk of reoperations should not be affected.

It can be argued that 1 or 2 years of experience perhaps is sufficient to be defined as an experienced surgeon due to the high number of hip fractures, and thus the 3-year limit is too high. Additionally, it is likely that the number of years of experience in fracture treatment does not necessarily reflect the volume of hip fracture operations that a surgeon has performed. Therefore, within both groups there can be some variation in how experienced the surgeons actually are in performing the different operation methods.

Another limitation was that experienced surgeons performed a majority of the procedures. The numbers managed by experienced surgeons was also increasing every year throughout the period we studied. Accordingly, it was difficult to find statistically significant differences in reoperations related to surgeons’ experience because the comparison basis was unbalanced. However, this trend is very positive in the surgical care of these elderly and frail patients.

In the NHFR, there is no information on radiological data. Accordingly, the quality of the primary operations (e.g., implant position and fracture reduction) cannot be assessed. Further, it is not possible to assess whether an eventual reoperation was caused by a new trauma or due to failure of an osteosynthesis.

Palm et al. (2007) did a prospective study including 600 patients with proximal femoral fracture and assessed the influence of surgeon’s experience and supervision on reoperation rate. In a multivariate analysis with patient demographics, their results showed unsupervised junior registrars to have a higher reoperation rate among what they defined as technically demanding procedures (primarily displaced femoral neck fractures and comminuted trochanteric fractures). Also, the group then introduced mandatory supervision for some procedures and a “driver’s license” for others (Palm et al. 2012). This is in accordance with our results.

Shervin et al. (2007) reviewed existing literature on the association between surgeon procedure volume and improved patient outcomes in orthopedic surgery. Their result suggested an association between higher surgeon volume and lower rates of hip dislocation. In addition, they found that surgeon volume was strongly related to revision of arthroplasties.

Khunda et al. (2013) reviewed the records of 761 patients who underwent surgery for proximal femoral fracture, to determine whether surgeon’s experience and volume was associated with 6-month mortality and reoperation rate. They concluded that the mortality rate within 6 months was 80% higher in patients operated by inexperienced surgeons. However, the patients operated by inexperienced surgeons were older, and the fractures were generally more complex in the consultant group, which could be a considerable weakness of that study. They found no association between increased reoperation risk and surgery performed by inexperienced surgeons.

Browne et al. (2009) and co-workers did a retrospective cohort study, including 97,894 patients surgically treated for hip fracture. The study addressed whether surgeon’s volume was associated with mortality or nonfatal morbidity. The mortality rate and incidence of transfusion, pneumonia, and decubitus ulcer was higher for patients managed by surgeons with low volume (< 7 procedures/year). Further, operations performed by low-volume surgeons were associated with nonfatal morbidity and longer hospital stay.

In the present study, the mortality was similar for patients managed by experienced and inexperienced surgeons. However, more patients with severe comorbidity (ASA 3 or ASA 4) and more patients with complex fractures were managed by experienced surgeons, and even if comorbidity and fracture type were adjusted for, there is a possibility that these differences still might have influenced the results.

Bjorgul et al. (2011) did a prospective study, including 1,780 hip fracture procedures. Their aim was to identify and characterize learning curves in hip fracture surgery. The results showed that mean operating time decreased for 4 different surgical procedures, though at different rates. This indicated unique learning curves for the 4 different procedures. This is in contrast to our findings where the durations of surgery for experienced surgeons compared with inexperienced surgeons were similar for hemiarthroplasty and longer for long IM nail. A possible explanation for this might be that the operations performed by experienced surgeons were more technically demanding. Less technically demanding operations, such as screw osteosyntheses and sliding hip screws, had a shorter operating time when performed by experienced surgeons.

### Explanations and interpretations

Few studies have addressed surgical experience and reoperation risk, and they lack consensus. We found an association between low experience with fracture surgery and higher reoperation risk after hemiarthroplasty and screw osteosynthesis for displaced FNFs. A similar increased risk for reoperation could not be found for undisplaced FNFs. An explanation could be that inexperienced surgeons do not manage to reduce the displaced FNFs properly or have problems in positioning the osteosynthesis material correctly when treating these fractures with osteosynthesis.

Correct anteversion of the femoral stem and correct leg length are factors important for stability of a hemiprosthesis. Experience is required to assess this correctly intraoperatively. A national registry can find otherwise hidden associations, e.g., between surgical inexperience and a higher reoperation rate, which merits new clinical studies with a higher degree of details such as, e.g., exact surgical technique and optimal reduction.

Today, surgeons with low experience perform a considerable proportion of hip fracture operations in Norway. The differences in risk of reoperations we found imply that experienced surgeons should manage some hip fracture types and operation methods. Based on our results the number needed to harm (NNH) for displaced FNFs was 25 (i.e., surgery performed by inexperienced surgeons resulted in 1 extra reoperation for every 25 operations compared with operations performed by experienced surgeons). If junior surgeons perform surgery for displaced femoral neck fractures, supervision by more experienced surgeons should be mandatory. For less technically demanding operations, junior surgeons could operate alone after they have obtained a “driver’s license” for that procedure, as described by Palm et al. (2012).

## Conclusion

Our findings suggest that surgeon’s experience has an impact on the risk of reoperation, in particular in some fracture types and operation methods. Experienced surgeons should manage or supervise all displaced femoral neck fractures, regardless of operation type, and all hemiprostheses for hip fractures.

The authors would like to thank the orthopedic surgeons in Norway for loyally reporting data on hip fracture operations to the NHFR.

Our study was planned and designed by ALA and JEG. ED and JEG performed the statistical analyses. ALA wrote the manuscript. All authors participated in the interpretation of data, and critical revision of the manuscript. 

*Acta* thanks Henrik Palm and Martyn Parker for help with peer review of this study.

## Supplementary Material

Supplemental Material
