# Supplementary material for: Surgeon’s experience level and risk of reoperation after hip fracture surgery: an observational study on 30,945 patients in the Norwegian Hip Fracture Register 2011–2015
Source: Acta Orthop. 2018 Jun 4;89(5):496–502. doi: 10.1080/17453674.2018.1481588 (PMC6202762; doi:10.1080/17453674.2018.1481588)
Supplement: Supplemental Material [file IORT_A_1481588_SM9035.pdf]

Supplementary data

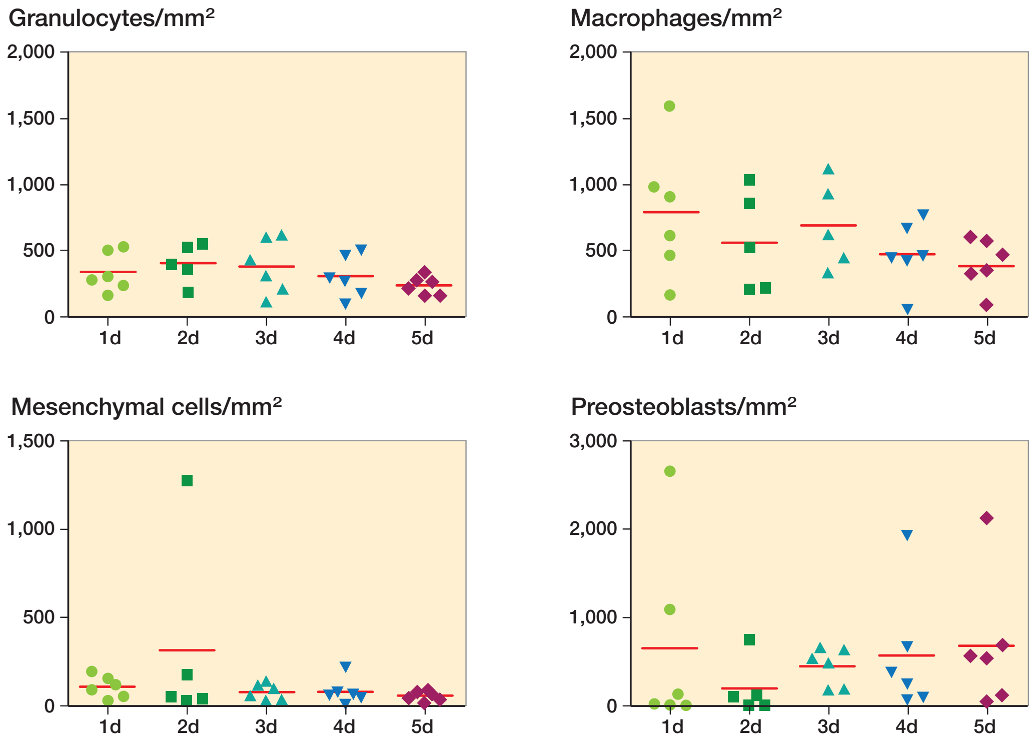

Figure 5. Quantification of cell populations in intact marrow in proximal tibia. No difference in cell numbers could be seen over the days for either of the markers.
